# Supplementary material for: Simultaneous inhibition of PI3K and PAK in preclinical models of neurofibromatosis type 2-related schwannomatosis
Source: Oncogene. 2024 Feb 9;43(13):921–30. doi: 10.1038/s41388-024-02958-w (PMC10959746; doi:10.1038/s41388-024-02958-w)
Supplement: Supplementary file 1 — Supplementary Material [file 41388_2024_2958_MOESM1_ESM.pdf]

Supplementary Tables

Supplementary Table 1. Targeted drug screen candidates for PI3K/Akt combination treatments and respective inhibited pathways.

Inhibitor	Target	Inhibitor	Target
Omipalisib	PI3Kα/β/δ/γ	Mebendazole	Tubulin depolymerization
		Ibrutinib	Brutons tyrosine kinase BTK & ITK
		Crizotinib	Multi-tyrosine kinase, c-MET
		PF-3758309	PAK4
		KPT-9274	PAK4 and NAMPT
		(-)-β-Hydrastine	PAK4
Pictilisib	PI3Kα/δ	PF-3758309	PAK4
Buparlisib	PI3Kα/β/δ/γ	Danusertib	Aurora-A/B/C Kinase
		Nintedamib	WGFR-1/2/3
		Crizotinib	Multi-tyrosine kinase, c-MET
		PF-3758309	PAK4
		KPT-9274	PAK4 and NAMPT
		(-)-β-Hydrastine	PAK4

Supplementary Table 2. EC50 drug concentrations for human (HS01, HS02/HS03) and mouse (MS01) MD-SC lines when drugs are delivered as monotherapies.

Inhibitor	EC50 HS01	EC50 HS02/HS03	EC50 MS01
Omipalisib	2.02 nM	26.8 nM	7.01 nM
Pictilisib	> 10 μM	> 10 μM	1462 nM
Buparlisib	1905 nM	2098 nM	870 nM
Mebendazole	2068 nM	774 nM	195 nM
Ibrutinib	7812 nM	11.7 μM	7045 nM
Crizotinib	1536 nM	621 nM	
PF-3758309	2009 nM	1765 nM	207 nM
KPT-9274	> 10 μM	691 nM	
(-)-β-Hydrastine	> 10 μM	384 nM	
Danusertib	1527 nM	15.6 μM	9071 nM
Nintedamib	5352 nM	> 10 μM	3121 nM

Supplementary Table 3. Loewe synergy scores (sum_syn_ant) for drug combinations in human MD-SC lines (HS01, HS03) and mouse MD-SC lines (MS01). Drugs were tested in 10x10 combination matrices.

Inhibitor	Inhibitor	Loewe Synergy Score (HS01)	Loewe Synergy Score (HS03)	Loewe Synergy Score (MS01)
**Omipalisib**	Mebendazole	16.49	3.25	2.53
	Ibrutinib	12.65	1.02	3.27
	Crizotinib	12.12	7.02	
	PF-3758309	73.55	12.12	
	KPT-9274	1.00	6.89	
	(-)-β-Hydrastine	8.42	43.60	
**Pictilisib**	PF-3758309	79.75	58.85	93.12
**Buparlisib**	Danusertib	28.4	12.06	1.99
	Nintedamib	4.27	43.69	7.76
	Crizotinib	9.44	4.79	
	PF-3758309	36.66	9.44	
	KPT-9274	33.47	28.06	
	(-)-β-Hydrastine	1.46	9.44	

Supplementary Figures

Supplementary Figure S1

Pictilisib vs. PF-3758309

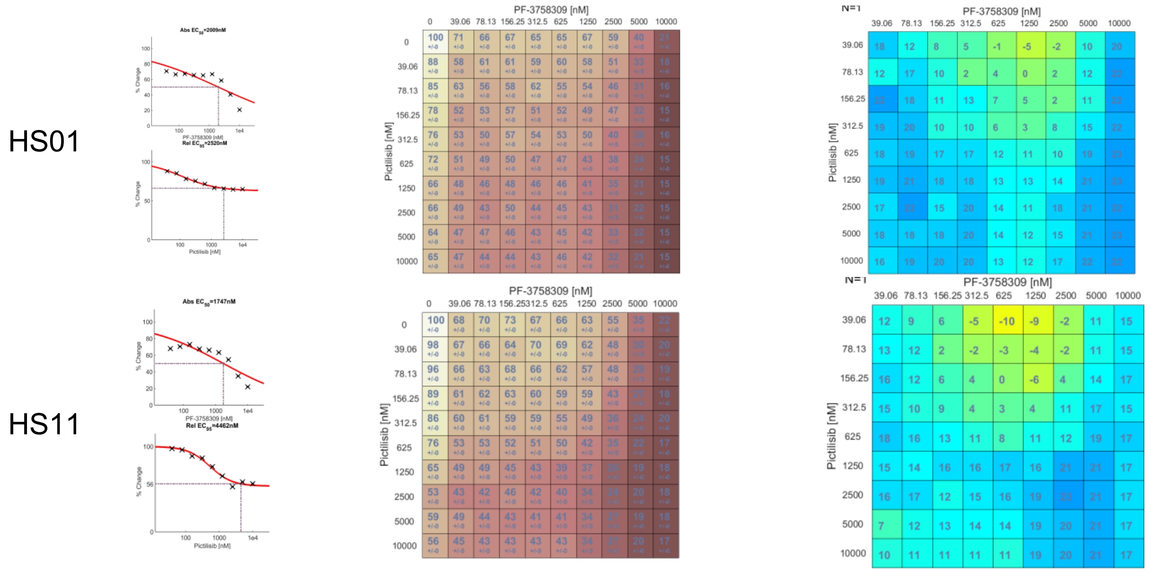

HS04.3

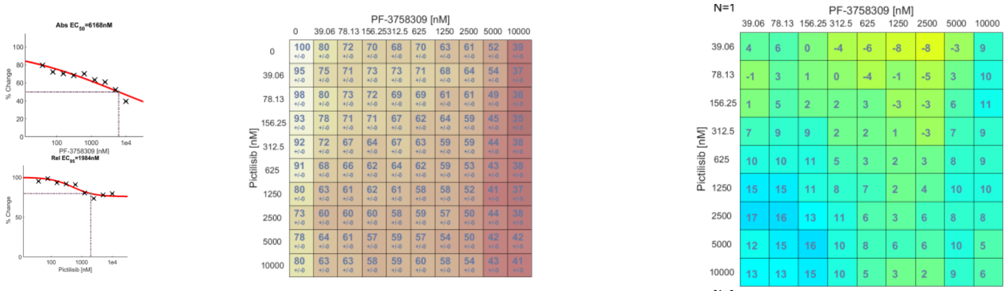

HS03

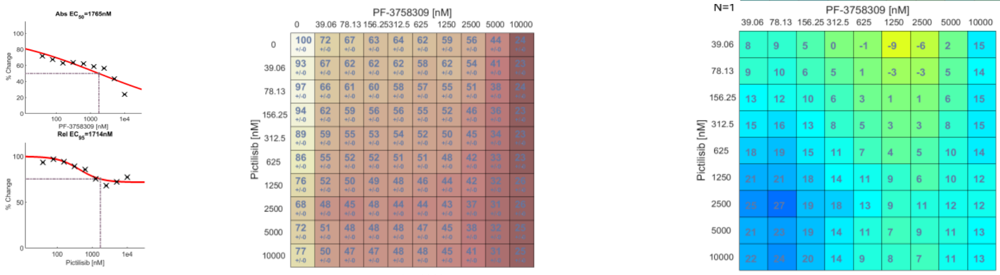

HS12

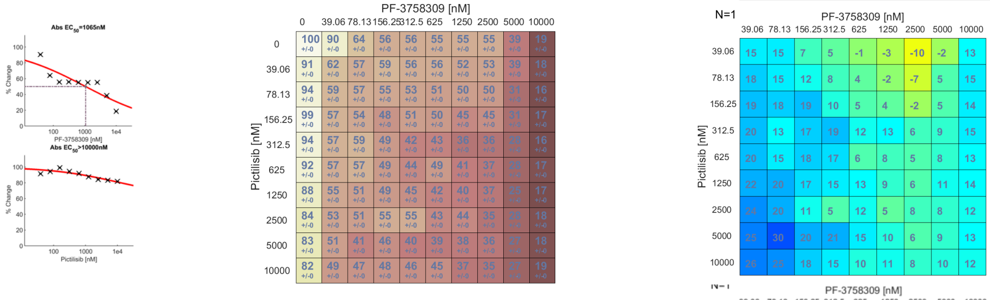

MS01

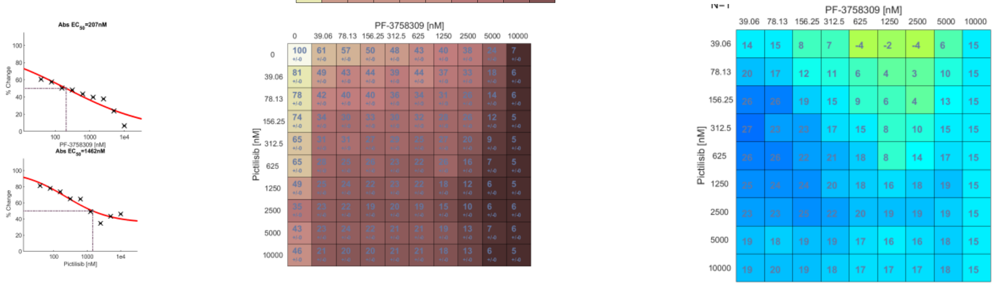

Buparlisib vs. Danusertib

HS01

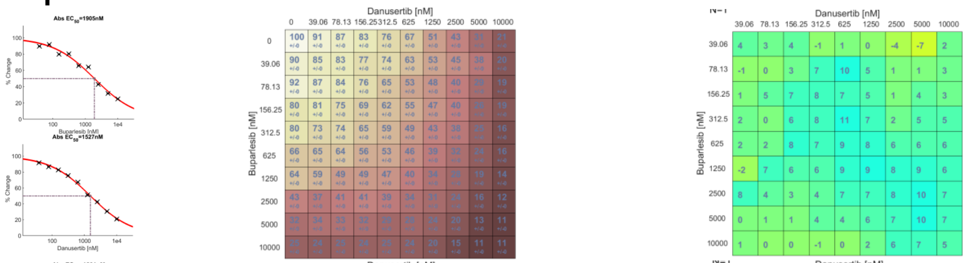

HS11

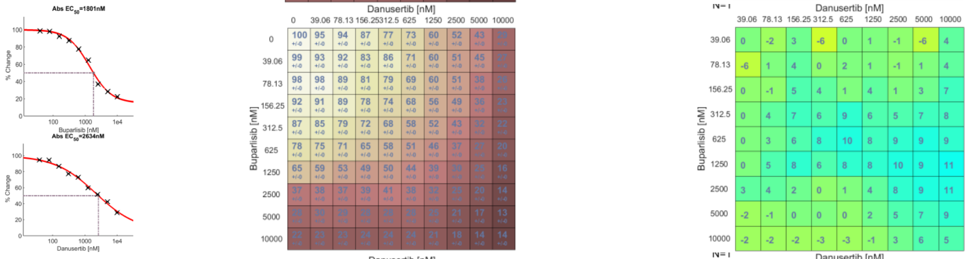

HS04.3

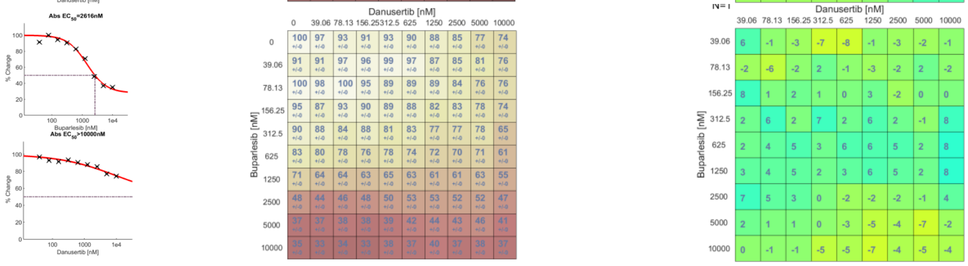

HS03

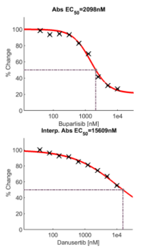

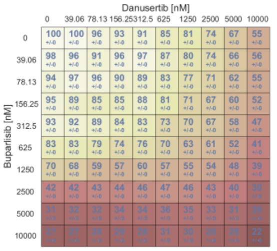

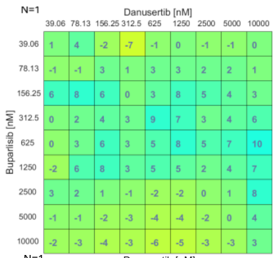

HS12

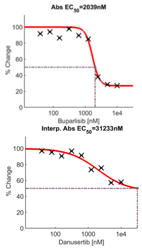

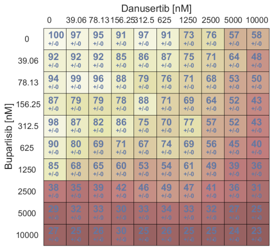

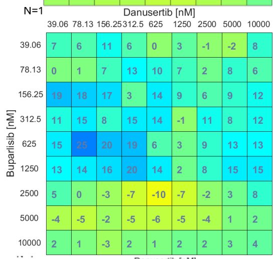

MS01

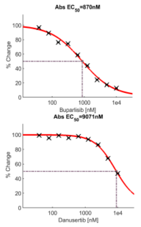

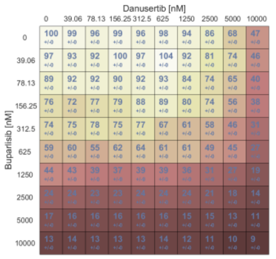

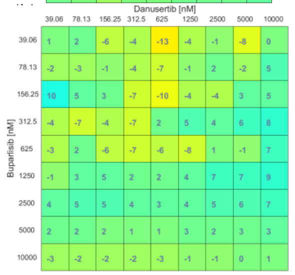

Buparlisib vs. Nintedamib

HS01

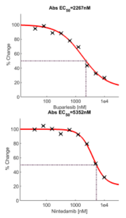

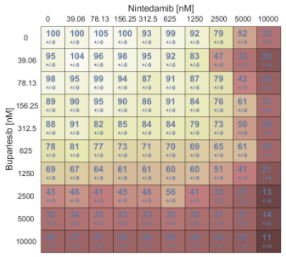

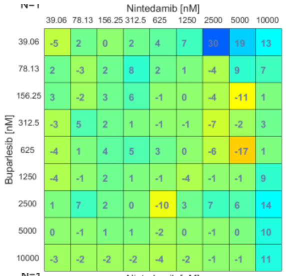

HS11

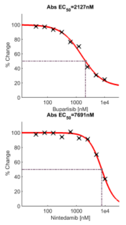

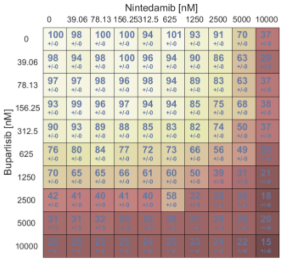

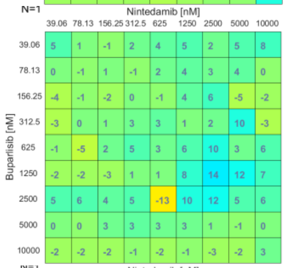

HS04.3

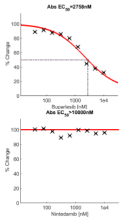

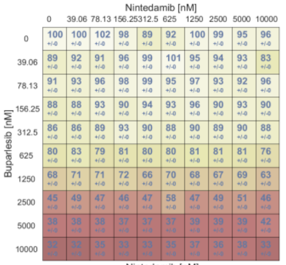

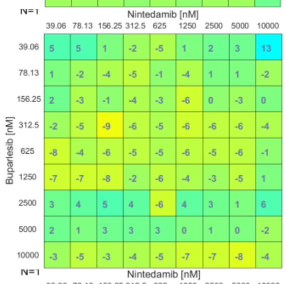

HS03

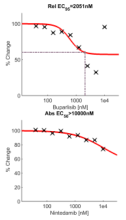

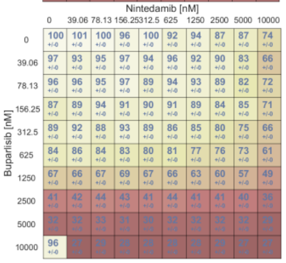

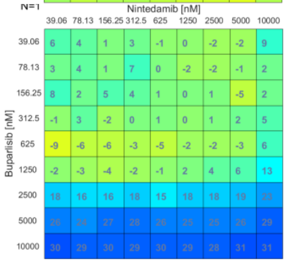

HS12

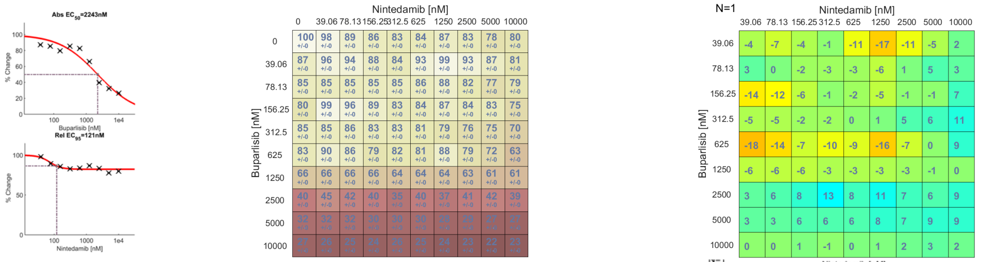

MS01

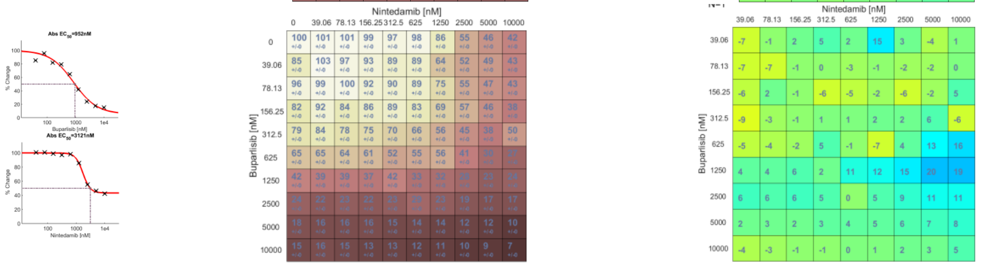

Omipalsib vs. TAE226

HS01

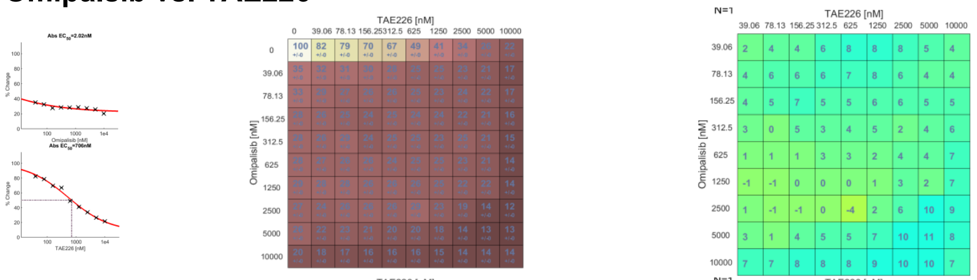

HS11

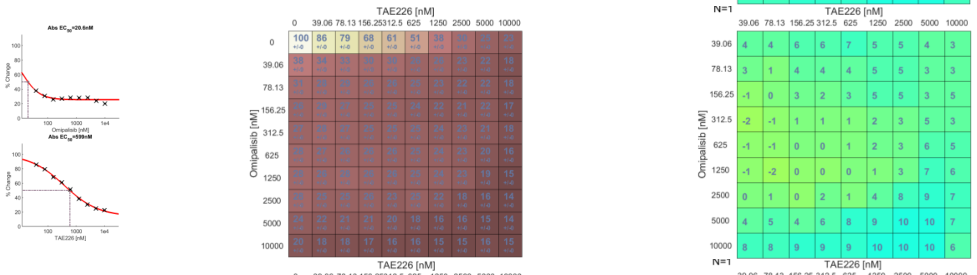

HS04.3

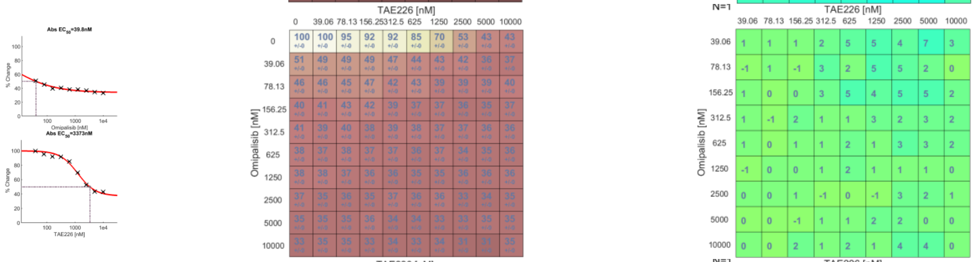

HS03

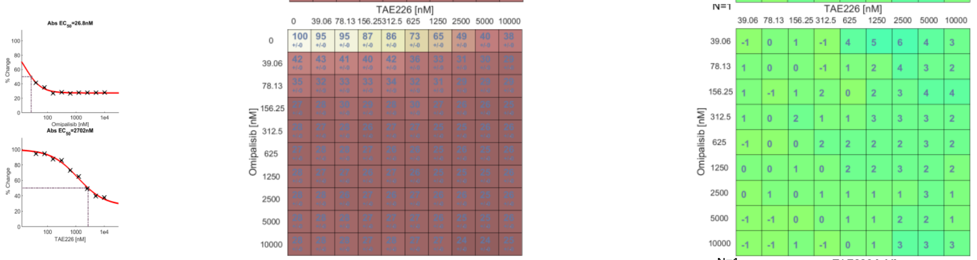

HS12

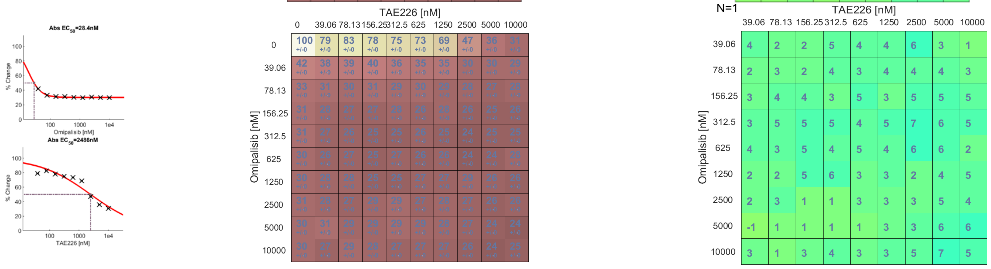

MS01

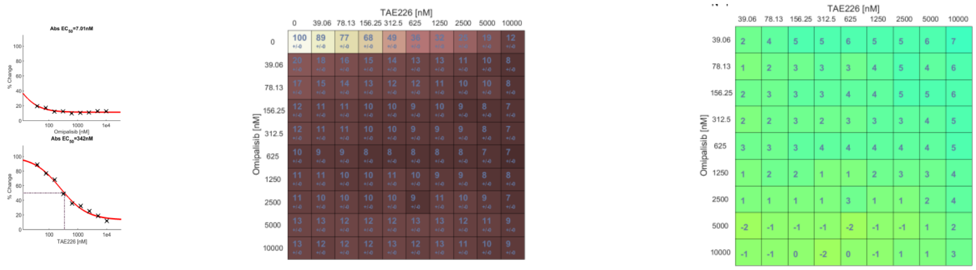

Omipalsib vs. Mebendazole

HS01

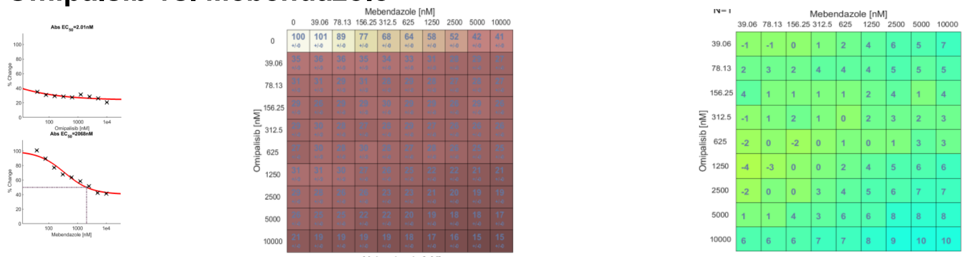

HS11

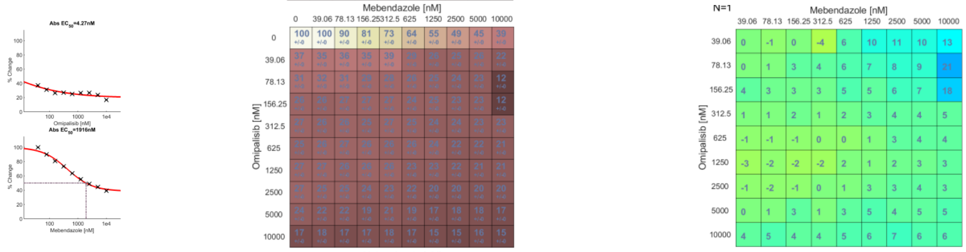

HS04.3

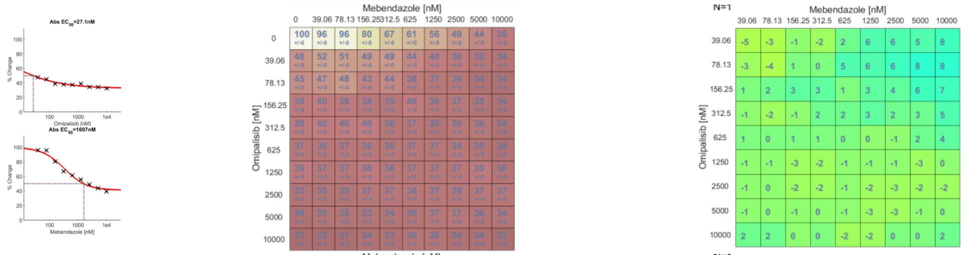

HS03

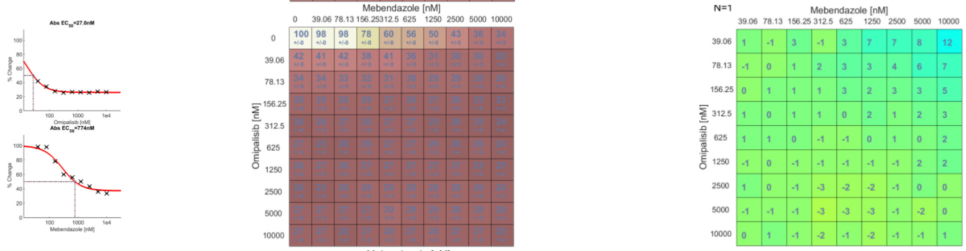

HS12

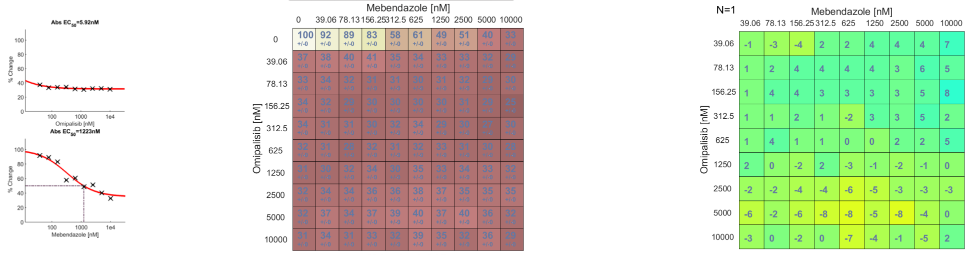

MS01

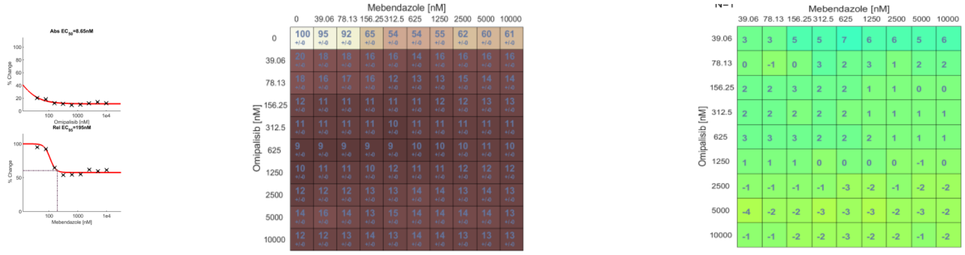

Omipalsib vs. Ibrutinib

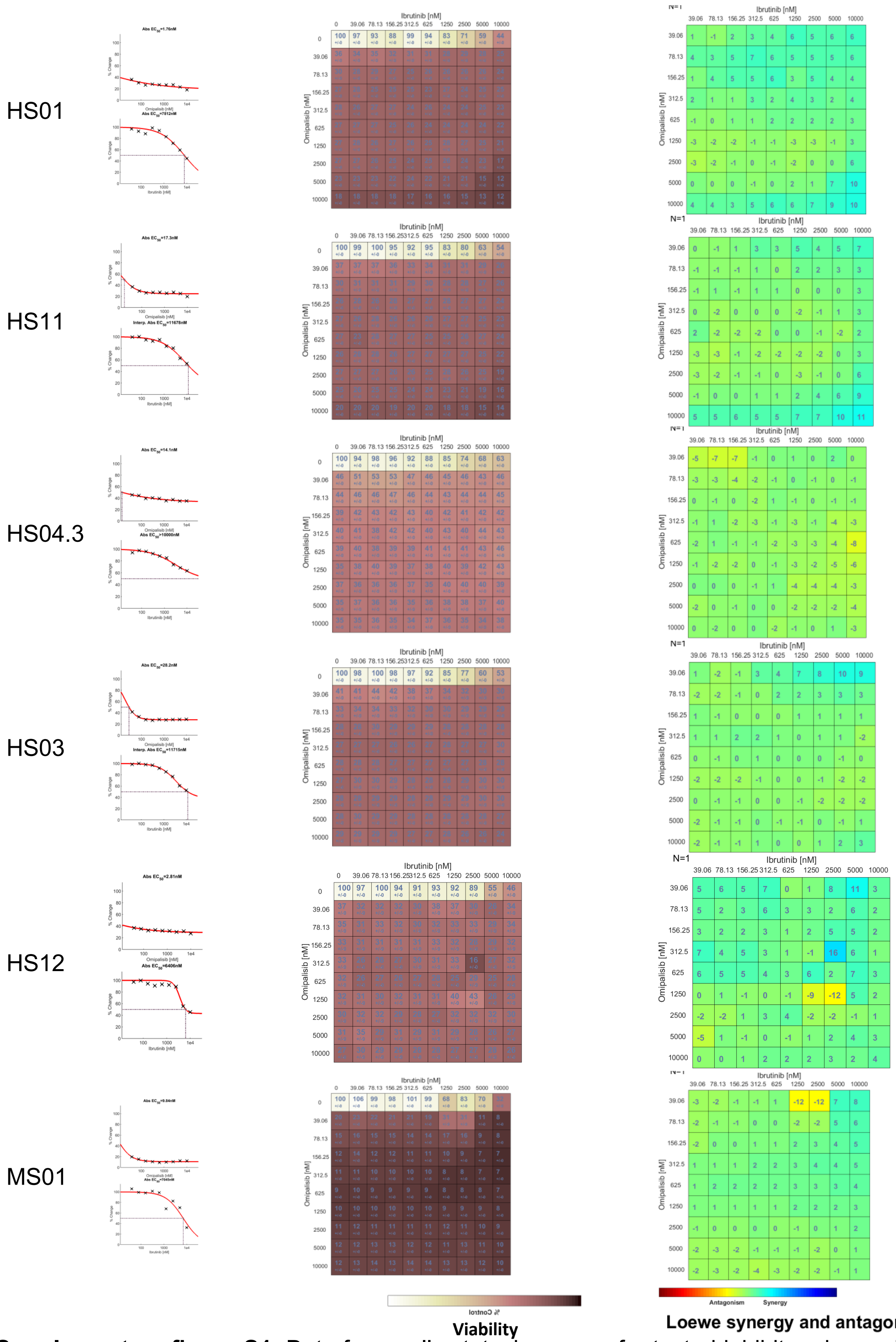

Supplementary figure S1. Data from cell cytotoxic assays for tested inhibitors in combinations by cell line: MS01 - mouse MD-SC line, HS01 – human MD-SC line (shRNA knock down), HS11 - human SC line, HS04.3 - human monoclonal MD-SC line (CRISPR/Cas9 knock out), HS03 - human MD-SC line (CRISPR/Cas9 knock out), HS12 - human Sc line.

Supplementary Figure S2

A

B

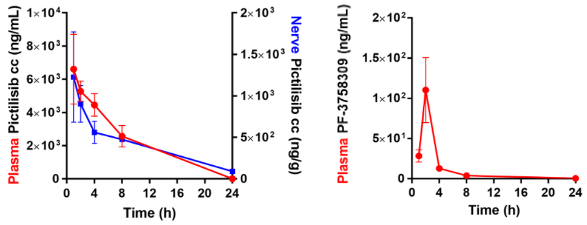

C

		Plasma			Nerve	
**Parameter**	**Unit**	**PF-3758309**	**Pictilisib**	**Unit**	**PF-3758309**	**Pictilisib**
t1/2	h	2.28	4.97	h	ND	16.51
Tmax	h	2.00	1.00	h	ND	1.00
Cmax	ng/ml	110.37	6609.33	ng/g	ND	1226.67

D

E

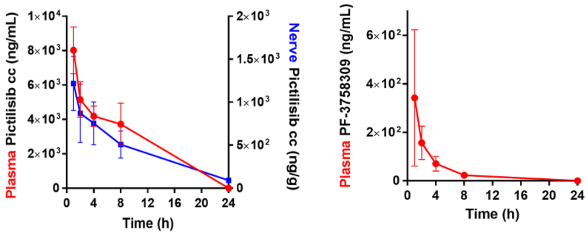

F

		Plasma			Nerve	
**Parameter**	**Unit**	**PF-3758309**	**Pictilisib**	**Unit**	**PF-3758309**	**Pictilisib**
t1/2	h	2.49	23.11	h	ND	7.06
Tmax	h	1.00	1.00	h	ND	1.00
Cmax	ng/ml	341.90	8017.00	ng/g	ND	1217.00

Supplementary Figure S2. Pharmacokinetic analysis of pictilisib and PF-3758309 as monotherapies and in combination after a single dose. (A) Plot of monotherapeutic pictilisib concentration as a function of time (plasma shown in red, nerve shown in blue). (B) Plot of monotherapeutic PF-3758309 concentration in plasma as a function of time (not detected in nerve). (C) Pictilisib and PF-3758309 pharmacokinetic parameters when delivered as monotherapeutics. (D) Plot of pictilisib concentration in the presence of PF-3758309 as a function of time (plasma shown in red, nerve shown in blue). (E) Plot of PF-3758309 concentration in plasma in the presence of pictilisib as a function of time (not detected in nerve). (F) Pictilisib and PF-3758309 pharmacokinetic parameters when delivered in combination. Data in graphs shown as mean±SEM. t1/2: plasma/nerve half-life; Tmax: time to maximum plasma/nerve concentration; Cmax: peak plasma/nerve concentration

Suuplementary Figure S3

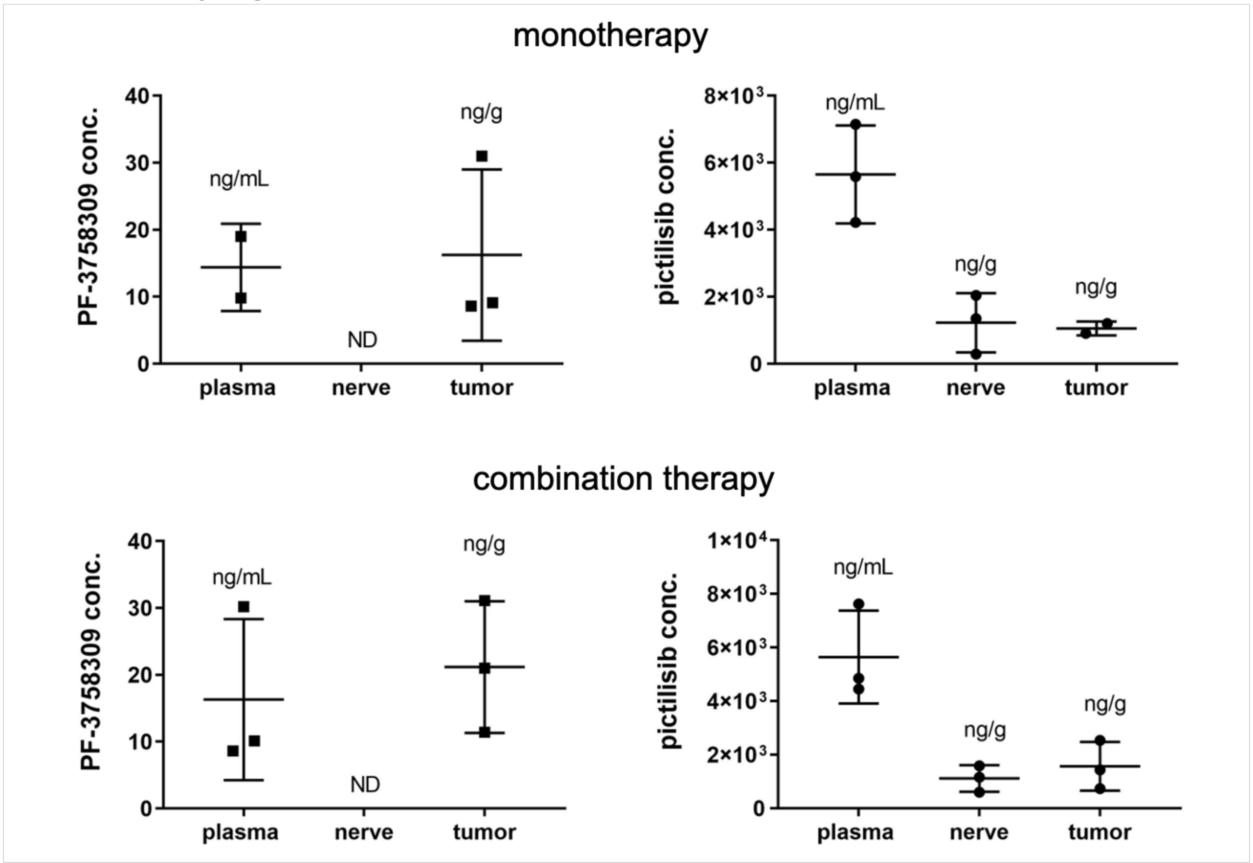

Supplementary Figure S3. Pharmacokinetic analysis of pictilisib and PF-3758309 as monotherapies and in combination after two weeks of daily dosing. PF-3758309, although not detected in naïve nerve, was detected in tumor, revealing disruption of the blood-nerve barrier in nerves grafted with the merlin-deficient Schwann cells and the possibility of reduced clearance rate from the tumors for PF-3758309. PF-3758309 was detected at similar levels in plasma and tumor when delivered alone and with pictilisib. Pictilisib was observed in the plasma, normal nerve and tumors of all mice treated as single or combination therapy.

Supplementary Figure S4

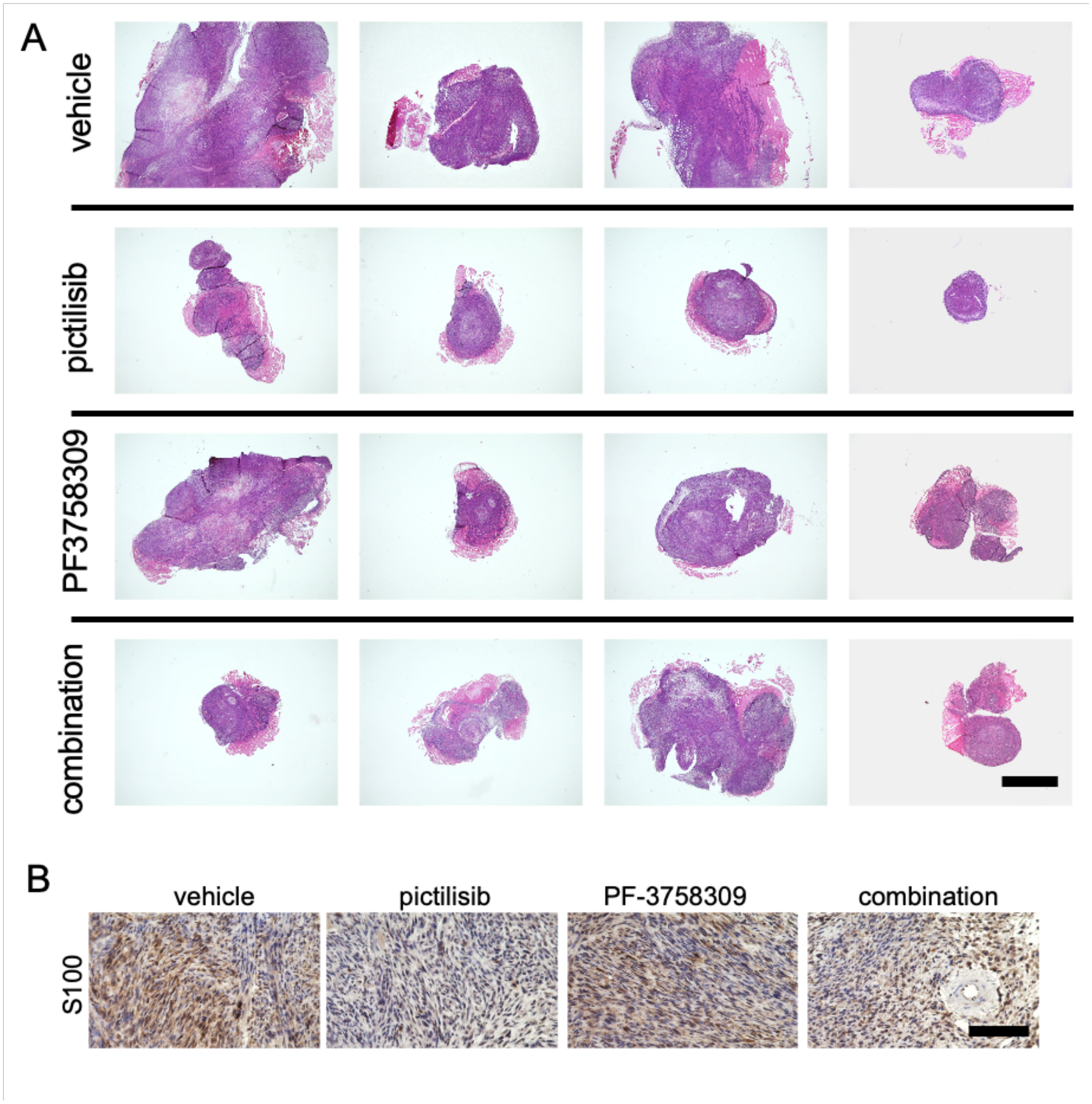

Supplementary Figure S4. Representative images of graft samples processed for histology (n=4/group) and (A) stained with hematoxylin and eosin imaged at 50x and (B) stained for S100 expression and imaged at 400x. Scale bar in A represents 1.5 mm. Scale bar in B represents 200 μm.

Supplementary Figure S5

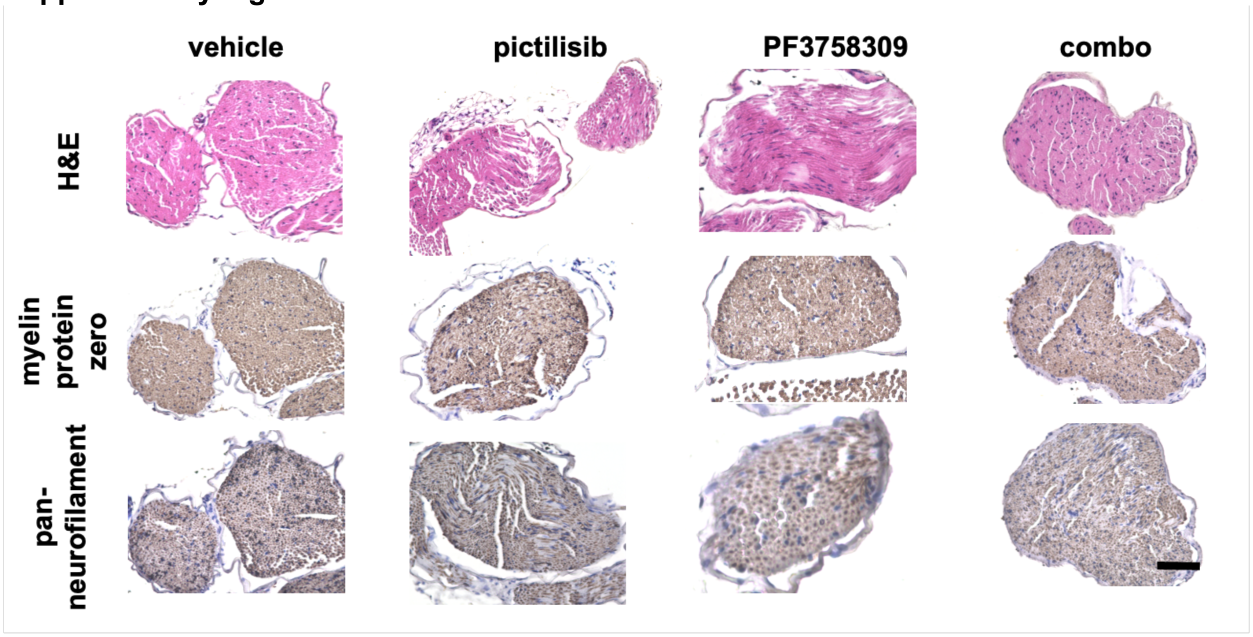

Supplementary Figure S5. Representative 100x images of contralateral control sciatic nerve samples processed for histology and stained with hematoxylin and eosin, myelin protein zero, and pan-neurofilament. There are no clear differences between groups in nerve morphology or Schwann cell-specific protein expression. Scale bar represents 400 µm.

Supplementary Figure S6

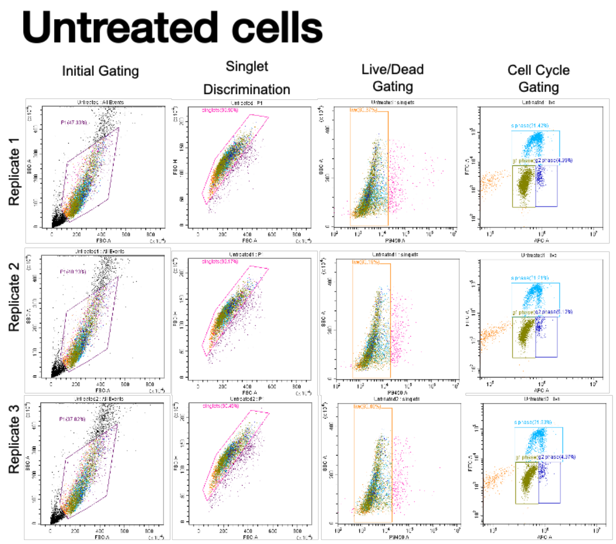

Tube Name: Untreated
Sample ID:

Population	Events...	% Total	% Pare...
All Events	20000	100.00%	100.00%
P1	9465	47.33%	47.33%
singlets	8893	44.47%	93.96%
live	8330	41.65%	93.67%
s phase	2617	13.09%	31.42%
g1 phase	4184	20.92%	50.23%
g2 phase	416	2.08%	4.99%

Tube Name: Untreated1
Sample ID:

Population	Events...	% Total	% Pare...
All Events	20000	100.00%	100.00%
P1	8045	40.23%	40.23%
singlets	7415	37.08%	92.17%
live	6910	34.55%	93.19%
s phase	2205	11.03%	31.91%
g1 phase	3451	17.26%	49.94%
g2 phase	354	1.77%	5.12%

Tube Name: Untreated2
Sample ID:

Population	Events...	% Total	% Pare...
All Events	20000	100.00%	100.00%
P1	7563	37.82%	37.82%
singlets	6844	34.22%	90.49%
live	6392	31.96%	93.40%
s phase	2041	10.21%	31.93%
g1 phase	3247	16.24%	50.80%
g2 phase	318	1.59%	4.97%

DMSO treated cells

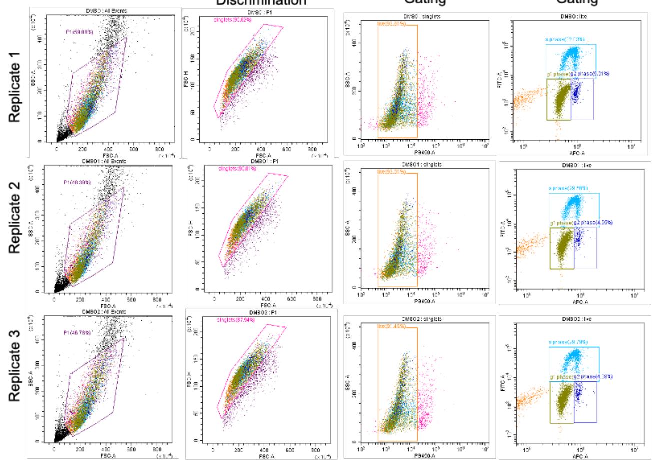

Tube Name: DMSO
Sample ID:

Population	Events...	% Total	% Pare...
All Events	20000	100.00%	100.00%
P1	11816	59.08%	59.08%
singlets	10708	53.54%	90.62%
live	10024	50.12%	93.61%
s phase	3211	16.06%	32.03%
g1 phase	5214	26.07%	52.02%
g2 phase	502	2.51%	5.01%

Tube Name: DMSO1
Sample ID:

Population	Events...	% Total	% Pare...
All Events	20000	100.00%	100.00%
P1	9677	48.39%	48.39%
singlets	8710	43.55%	90.01%
live	8101	40.51%	93.01%
s phase	2395	11.98%	29.56%
g1 phase	4295	21.48%	53.02%
g2 phase	369	1.85%	4.55%

Tube Name: DMSO2
Sample ID:

Population	Events...	% Total	% Pare...
All Events	20000	100.00%	100.00%
P1	9355	46.78%	46.78%
singlets	8227	41.14%	87.94%
live	7527	37.64%	91.49%
s phase	2242	11.21%	29.79%
g1 phase	3979	19.90%	52.86%
g2 phase	368	1.84%	4.89%

PF-3758309 treated cells

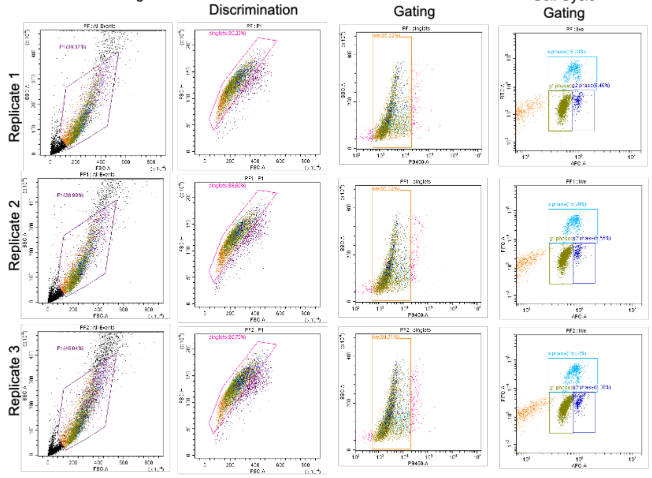

Tube Name: PF
Sample ID:

Population	Events...	% Total	% Pare...
All Events	20000	100.00%	100.00%
P1	7673	38.37%	38.37%
singlets	6923	34.62%	90.23%
live	6592	32.96%	95.22%
s phase	1004	5.02%	15.23%
g1 phase	3871	19.36%	58.72%
g2 phase	426	2.13%	6.46%

Tube Name: PF1
Sample ID:

Population	Events...	% Total	% Pare...
All Events	20000	100.00%	100.00%
P1	7798	38.99%	38.99%
singlets	6909	34.55%	88.60%
live	6565	32.83%	95.02%
s phase	998	4.99%	15.20%
g1 phase	3944	19.72%	60.08%
g2 phase	430	2.15%	6.55%

Tube Name: PF2
Sample ID:

Population	Events...	% Total	% Pare...
All Events	20000	100.00%	100.00%
P1	9007	45.04%	45.04%
singlets	7814	39.07%	86.75%
live	7401	37.01%	94.71%
s phase	1172	5.86%	15.84%
g1 phase	4594	22.97%	62.07%
g2 phase	620	3.10%	8.38%

Pictilisib

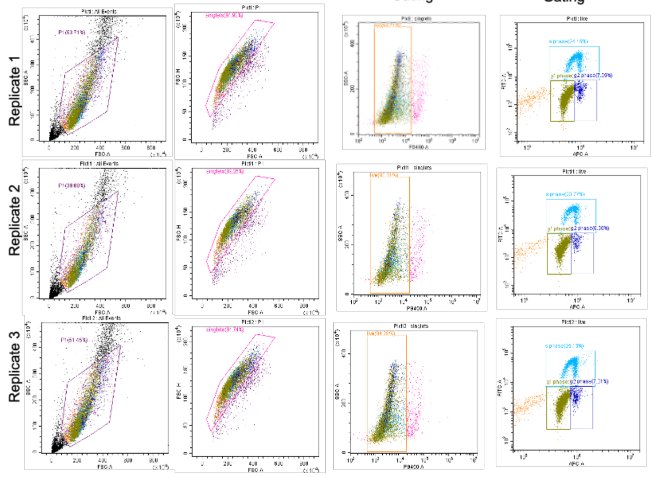

Tube Name: Picti
Sample ID:

Population	Events...	% Total	% Pare...
All Events	20000	100.00%	100.00%
P1	10741	53.71%	53.71%
singlets	9880	49.40%	91.98%
live	9357	46.79%	94.71%
s phase	2260	11.30%	24.15%
g1 phase	5828	29.14%	62.28%
g2 phase	663	3.32%	7.09%

Tube Name: Picti1
Sample ID:

Population	Events...	% Total	% Pare...
All Events	20000	100.00%	100.00%
P1	7815	39.08%	39.08%
singlets	6975	34.88%	89.25%
live	6543	32.72%	93.81%
s phase	1555	7.78%	23.77%
g1 phase	4084	20.42%	62.42%
g2 phase	412	2.06%	6.30%

Tube Name: Picti2
Sample ID:

Population	Events...	% Total	% Pare...
All Events	20000	100.00%	100.00%
P1	10290	51.45%	51.45%
singlets	9440	47.20%	91.74%
live	8901	44.51%	94.29%
s phase	2237	11.19%	25.13%
g1 phase	5420	27.10%	60.89%
g2 phase	651	3.26%	7.31%

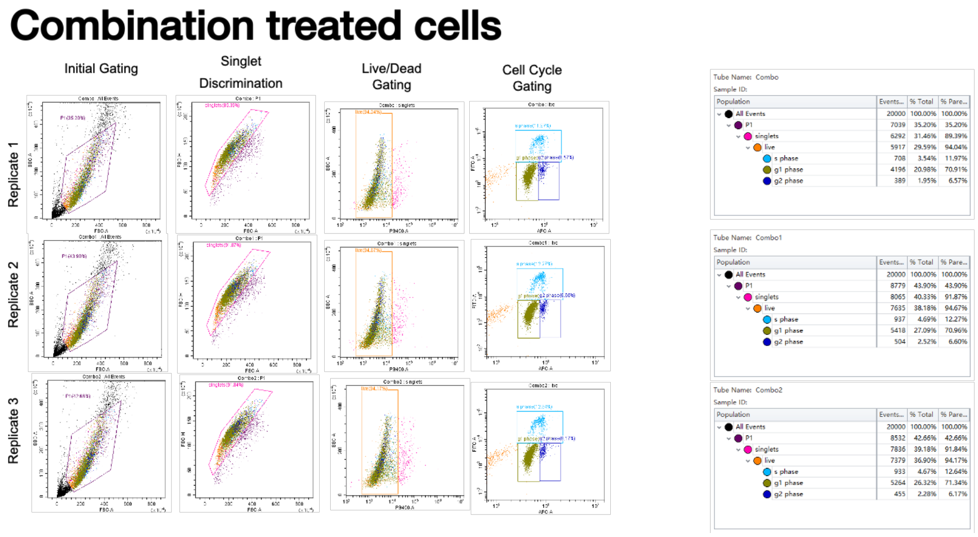

Tube Name: Combo
Sample ID:

Population	Events...	% Total	% Pare...
All Events	20000	100.00%	100.00%
P1	7039	35.20%	35.20%
singlets	6292	31.46%	89.39%
live	5917	29.59%	94.04%
s phase	708	3.54%	11.97%
g1 phase	4196	20.98%	70.91%
g2 phase	389	1.95%	6.57%

Tube Name: Combo1
Sample ID:

Population	Events...	% Total	% Pare...
All Events	20000	100.00%	100.00%
P1	8779	43.90%	43.90%
singlets	8065	40.33%	91.87%
live	7635	38.18%	94.67%
s phase	937	4.69%	12.27%
g1 phase	5418	27.09%	70.96%
g2 phase	504	2.52%	6.60%

Tube Name: Combo2
Sample ID:

Population	Events...	% Total	% Pare...
All Events	20000	100.00%	100.00%
P1	8532	42.66%	42.66%
singlets	7836	39.18%	91.84%
live	7379	36.90%	94.17%
s phase	933	4.67%	12.64%
g1 phase	5264	26.32%	71.34%
g2 phase	455	2.28%	6.17%

Supplementary Figure S6. Flow analysis of cell cycle in treated and untreated HS01 cells.

Supplementary Figure S7

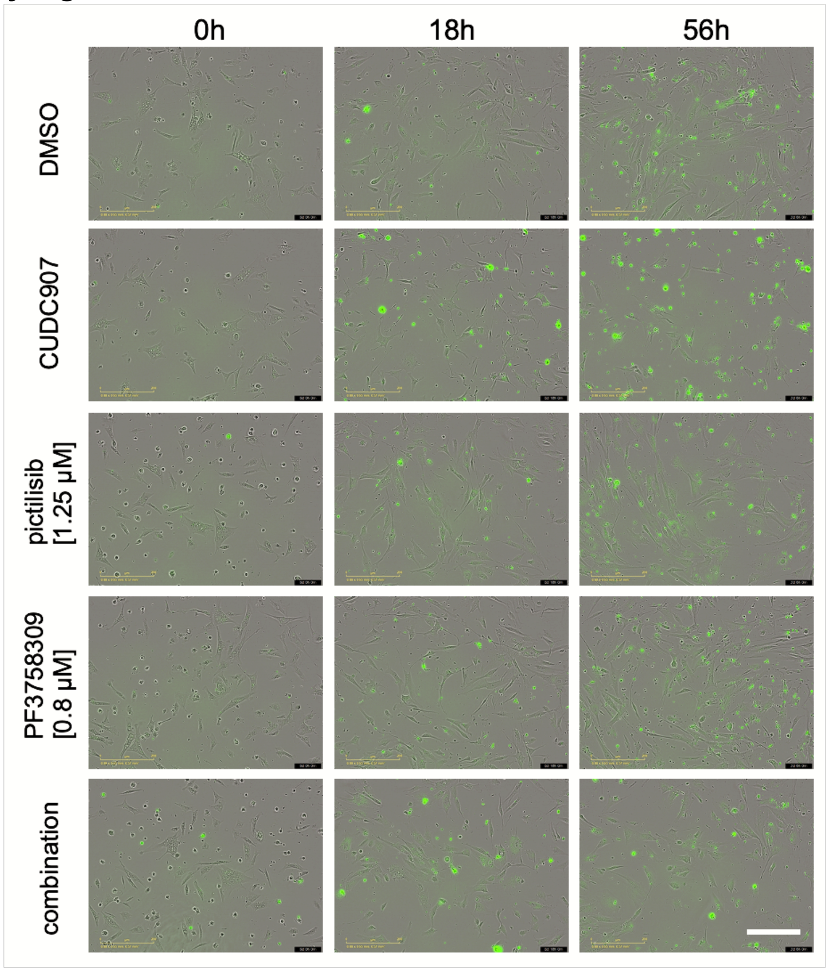

Supplementary Figure S7. Representative images of cleaved caspsase3/7+ (green) cells imaged at 0h, 18h, and 54h of cultures with indicated treatments.

Supplementary Figure S8.

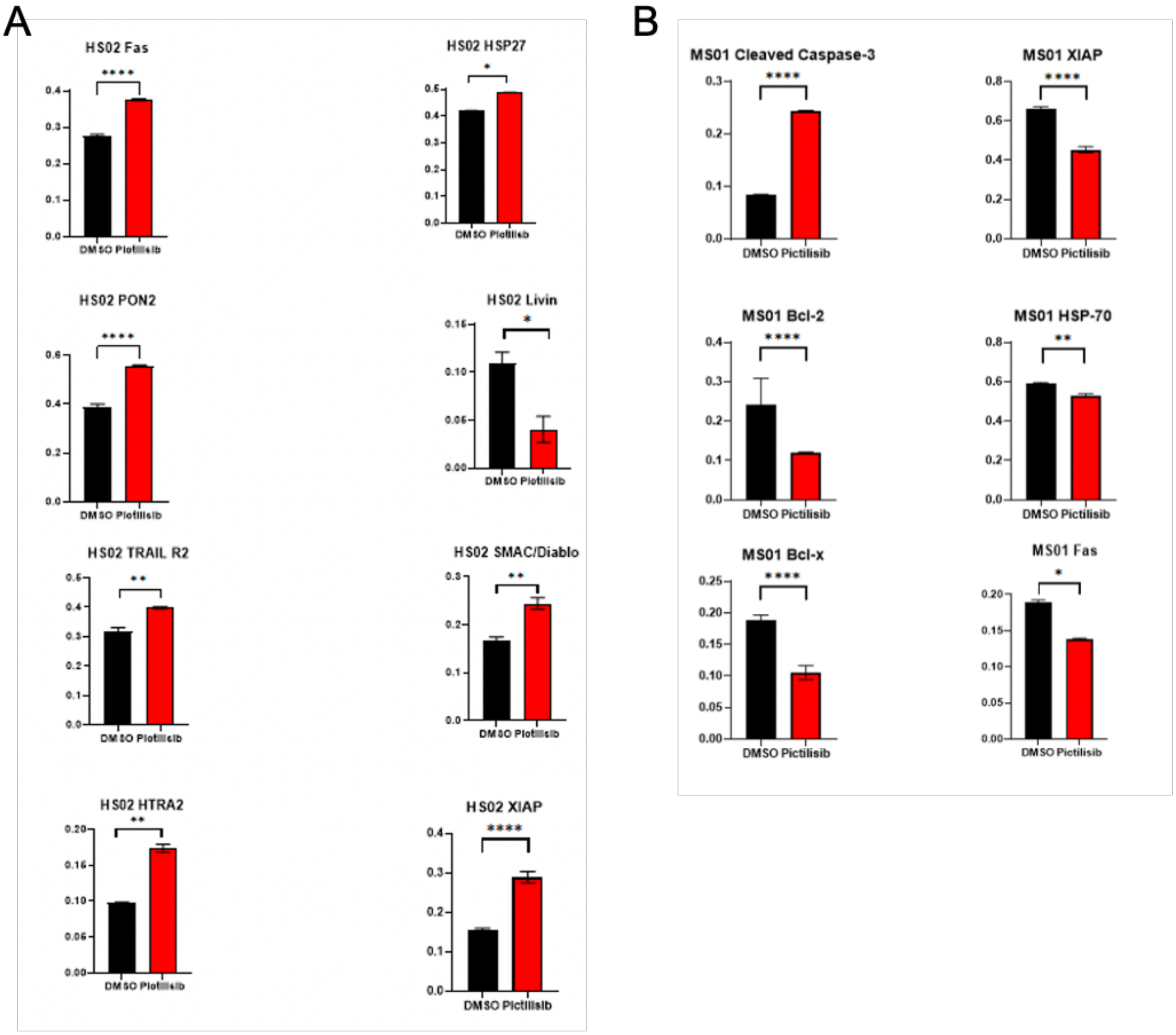

Supplementary Figure S8. All significant differences in protein levels detected by human (A) and mouse (B) Apoptosis Proteome Profiles arrays. *p<0.05; **p<0.01, ****p<0.0001 by Šídák's multiple comparisons test.
